# Clinical Course of Hashimoto's Thyroiditis and Effects of Levothyroxine Therapy on the Clinical Course of the Disease in Children and Adolescents

**DOI:** 10.4274/jcrpe.425

**Published:** 2011-12-06

**Authors:** Samim Özen, Ömer Berk, Damla Gökşen Şimşek, Şükran Darcan

**Affiliations:** 1 Mersin Children’s Hospital, Pediatric Endocrinology Unit, Mersin, Turkey; 2 Ege University, School of Medicine, Department of Pediatrics, Izmir, Turkey; 3 Ege University, School of Medicine, Department of Pediatric Endocrinology, Izmir, Turkey; +90 324 223 07 01 / 435 +90 324 223 07 22samimozen@gmail.comMersin Children’s Hospital, Pediatric Endocrinology Unit, 33240 Güneykent, Mersin, Turkey

## Abstract

**Objective:** The aim of this study was to evaluate the clinical course of   Hashimoto’s thyroiditis (HT) in children and adolescents and the effects of levothyroxine therapy on the clinical course and laboratory findings.

**Methods:** The clinical and laboratory data of 101 patients with HT  at presentation and during a three-year follow-up period were retrospectively evaluated using patient records.

**Results:** The mean age of the patients at the time of diagnosis was 12.3±2.90 years  and female/male ratio was  5.7/1. The complaint at the time of hospital presentation was goiter in 57.8% of the patients. At baseline, 36.7% of the patients were euthyroid, whereas 32.7% had subclinical hypothyroidism, 16.6 % of subjects were evaluated as hypothyroid. Twelve of the 28 patients who were initially euthyroid and not receiving therapy developed subclinical or overt hypothyroidism during the first 18 months of the follow-up period and were started on thyroid medication. At presentation, the mean anti-thyroglobulin (anti-Tg) and anti-thyroperoxidase antibody levels were 450±725 IU/mL and 392±428 IU/mL, respectively and at the end of the follow-up period, a significant decrease was observed in the anti-Tg levels of patients receiving levothyroxine from the beginning.

**Conclusions:** Thyroid functions of the patients with HT should be monitored periodically for hypothyroidism. Levothyroxine therapy may positively affect the clinical course of the disease and the antibody titers.

**Conflict of interest:**None declared.

## INTRODUCTION

Hashimoto's thyroiditis (HT), also known as chronic lymphocytic thyroiditis or chronic autoimmune thyroiditis, is the most common form of thyroiditis in childhood (1,2). The pathophysiology of the disease can be summarized as follows: triggering of humoral immunity by the abnormal stimulation of T-lymphocytes and consequent destruction of thyroid epithelial cells by chemotaxis, autoantibodies and inflammatory cascade. The degradation of the cells may be compensated by the increased thyroid-stimulating hormone (TSH) levels and the hyperplasia of epithelial cells. If not treated, HT may lead to retardation in growth and development, resulting in short stature, decline in school, performance, and anemia (1,2,3,4,5,6,7,8).

Studies regarding the clinical course of the disease in childhood are quite limited. Discussions are mainly focused on whether subclinical hypothyroidism should be treated or not. Although there are accepted criteria for the treatment of HT in adults, there are no generally accepted treatment guidelines for children. Nonetheless, many pediatric 

endocrinologists believe that subclinical hypothyroidism should be treated in childhood, at a time when growth and development have not yet been completed. However, the number of studies investigating  the effect of therapy on the clinical course of the disease in childhood is  limited (2,4,6,7,8).

The aim of the present study was to observe the clinical course of HT in children and adolescents and to determine the effects of levothyroxine treatment on the clinical course, on antibody titers and on growth.  

## MATERIALS AND METHODS

The outpatient records of 101 children and adolescents younger than 18 years of age, who presented to the Pediatric Endocrinology Outpatient Clinic of Ege University School of Medicine between 2002 and 2006, were evaluated retrospectively. HT patients who had regularly attended the scheduled visits for at least two years were included in 

the study. Patients who had a history of a syndrome or chromosome abnormality, premature birth or any chronic disease were excluded.  The diagnosis of HT was based on ultrasonography (US) and laboratory criteria, and mainly on detection of antithyroid antibodies. Free triiodothyronine (fT3), free thyroxine (fT4), TSH,  anti-thyroperoxidase 

(anti-TPO) and anti-thyroglobulin (anti-Tg) antibody levels were measured. In patients with a diagnosis of HT,  in addition to clinical and US findings, the anti-TPO antibody titers were greater than 200 IU/mL.  Age and gender of the patients, as well as their complaints at the time of presentation, family history of thyroid disease, concomitant diseases, and birth weight were recorded. The anthropometric measurements, pubertal stage and physical examination findings of the patients, their antithyroid antibody levels, results of thyroid imaging studies, drug treatment regimen, fT3, fT4 and TSH levels and anti-TPO and anti-Tg antibody levels at presentation and during follow-up were also 

recorded. The relationship between antibody levels and thyroid hormone replacement therapy was evaluated. The findings were analyzed by classifying the data as "data at presentation", "data in the 1st follow-up period" (6-18 months), and "data in the 2nd follow-up period" (18-36 months). Height, weight, and body mass index (BMI) standard deviation scores (SDS) were calculated according to the reference values defined for Turkish children by Neyzi et al (9).Laboratory analysis of fT3, fT4 and TSH levels was done with commercial test kits (Roche Cobas®) using Elecsys 2010® analyzer every 3-6 months. The corresponding normal values for the 2.5th and 97.5th percentiles of fT3, fT4 and TSH were 2.0-4.4 pg/mL, 0.93-1.7 ng/dL, and 0.27-4.2 μIU/mL, respectively. The patients were evaluated as euthyroid, subclinical hypothyroid, hypothyroid, subclinical hyperthyroid, or hyperthyroid,  according to their fT4 and TSH levels. The HT subjects were classified to have subclinical hypothyroidism if they had an elevated serum TSH (TSH >4.7 mIU/mL) and normal fT4 concentrations (fT4: 0.82-1.87 ng/dL). If an elevated serum TSH concentration was associated with a decreased fT4 concentration, the patient was considered as an overt hypothyroid. Those with low TSH and elevated thyroid hormone levels were accepted as cases of hyperthyroidism and those with low TSH and normal thyroid hormone levels as subclinical hyperthyroidism. Anti-Tg and anti-TPO antibody measurements were done using Immulite 2000® kits and Siemens® analyzer every 6-12 months. The 95th percentile values for anti-Tg and anti-TPO antibody levels were 40 IU/mL and 35 IU/mL, respectively.

US examinations were performed by a radiologist, using high-frequency US. The echogenicity and the size of the thyroid gland, presence of thyroid nodules and the lymph nodes around the gland were evaluated. Thyroid gland volume was calculated using the formula proposed by Delange et al (10), by multiplying the height, width and thickness of both lobes of the gland with 0.479. The volume of the thyroid gland was compared with the reference values proposed by the World Health Organization in 1997 (11). 

Levothyroxine was initiated in patients with subclinical hypothyroidism, overt hypothyroidism and also in goitrous (on US) euthyroid patients. The changes in antibody titers on levothyroxine therapy, clinical response to the therapy, US examination findings during follow-up, and the relationship between dose increment and antibody titers were retrospectively investigated.

The study was reviewed and approved by the Ege University Ethics Committee. The authors confirmed in writing that they have complied with the World Medical Association Declaration of Helsinki regarding ethical conduct of research involving human subjects and/or animals.

## STATISTICAL ANALYSIS

After the data were transferred to digital media, Statistical Package for the Social Sciences (SPSS) for Windows (version 16.0; SPS Inc., Chicago, IL, USA) was used for analysis. Mann-Whitney U-test, repeated-measure test and the Kruskal-Wallis test were used for comparisons. Frequencies were compared using the Chi-square (χ2) test. A p-value of less than 0.05 was considered to be significant in all tests.

## RESULTS

Of the 101 patients included in the study, 86 were girls and 15 were boys. Female/male ratio was 5.7/1. The mean age of the patients at the time of diagnosis was 12.3±2.9 years (range: 4.4-17.9). The mean ages of the male and female patients at the time of diagnosis were 13.0±2.4 and 12.2±3.0 years, respectively. The most common complaint at resentation was swelling in the neck (58%), followed by nervousness (18%), dermatological problems (13%), and hair loss (8%).

Thyroid function impairment and/or goiter were present in the mother, father or siblings of 33% of male and 28% of female patients.

Height, weight and BMI SDS values of patients at presentation were 0.10±1.15, 0.01±1.30 and 0.08±1.15, respectively.

At presentation, the laboratory findings of 36.7% of the patients were consistent with euthyroidism, those of 32.7% with subclinical hypothyroidism. 16.6% of subjects were evaluated as hypothyroid, 7.9% as having subclinical hyperthyroidism, and 5.9% as having hyperthyroidism (Table 1). At presentation, the mean anti-Tg and anti-TPO antibody levels were 450±725 IU/mL and 392±428 IU/mL, respectively. 

**Results of the first follow-up period **

Of the 96 patients who continued to attend the clinic after the first follow-up period (350±137 days), 68 (70%) were receiving treatment, while 28 were being followed without treatment. Levothyroxine was discontinued in two patients during the follow-up, and five patients were lost to follow-up.

Anti-Tg and anti-TPO antibody values of the 28 cases who were not receiving treatment were 427±780 IU/mL and 257±374 IU/mL, respectively. The antibody levels of this group were compared with those of the group of 68 patients who were receiving treatment and were on regular follow-up from the beginning of the study. In both groups, anti-Tg antibody values decreased, whereas anti-TPO antibody values showed an increase (Table 2). The changes which occurred in anti-Tg and anti-TPO antibody values over the first year of follow-up showed no significant differences between the two groups (p=0.35 for anti-Tg and p=0.09 for anti-TPO). There also were no significant difference in terms of height, weight and BMI SDS changes between the two groups (p>0.05).

In our study, 68 of our 101 patients were receiving treatment at the end of the first follow-up period (6-18 months). Of these patients, seventeen were hypothyroid and 33 had subclinical hypothyroidism. Whereas 36.7% of patients who had been receiving therapy at presentation were euthyroid and 32.7% had subclinical hypothyroidism, these rates changed to 66.7% and 13.5%, respectively, after the first follow-up period (Table 3). 

The patients who were on thyroid treatment were receiving a mean levothyroxine dose of 1.82±0.205 mg/kg at the time of presentation and the dose was nearly the same (1.9±0.88 mg/kg) after the first follow-up period.

**Results of the second follow-up period**

Ninety-six patients in the first follow-up period continued to be followed in the second follow-up period. In this period, 12 (42.8%) of the 28 patients who were being followed-up without any treatment in the first follow-up period developed subclinical hypothyroidism and were started on thyroid treatment.  No statistically significant difference was found in terms of anti-Tg and anti-TPO antibody values and anthropometric measurements between these 12 patients who received treatment after the first follow-up period and 16 patients who continued their follow-up without any treatment. In the second follow-up period, the decrease in anti-Tg antibody values in  the treated group  was significant (p=0.01). However, the mean anti-TPO antibody values remained high despite therapy (Table 4).

No significant difference was found in terms of weight, height, and BMI SDS values between the treated group and the group not receiving therapy. 

Eighty-nine of 101 cases underwent US and thyromegaly was detected in 31 (34%) of them. Follow-up US was performed in 48 cases after two years. Eight of 18 patients, who had thyromegaly and were receiving treatment at presentation, underwent follow-up US, and the size of the thyroid gland was found to be decreased or reverted to normal size in 7 of these 8 patients.

No remarkable decrease in the size of the thyroid gland was found in the group not receiving therapy. Thyromegaly was observed in the repeated US of three cases that did not have thyromegaly initially.

**Table 1:**
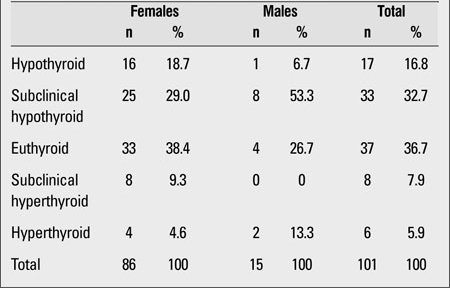
Thyroid status of the patients at presentation

**Table 2:**
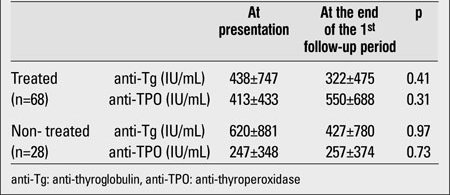
Comparison of antibody levels in the treated and non-treated groups at the end of the first follow-up period

**Table 3:**
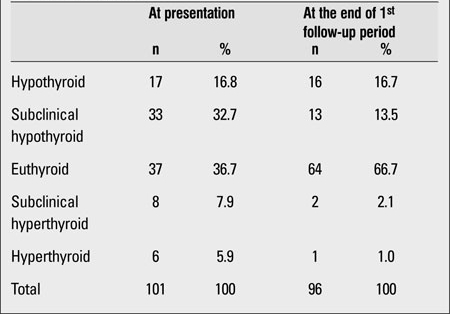
Thyroid status of the patients at presentation and at the end of the first follow-up period

**Table 4:**
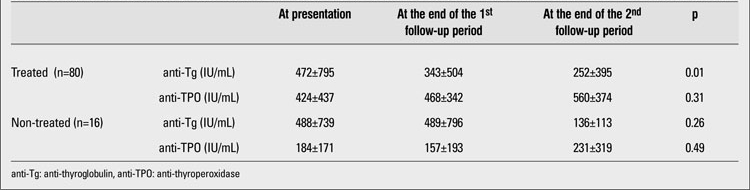
Comparison of antibody levels in the treated and non-treated groups at presentation and at the end of the first and second follow-up periods

## DISCUSSION

Although autoimmune thyroiditis in adults has been extensively investigated, studies on children and adolescents are limited. The natural course of juvenile autoimmune hyroiditis is quite variable (8,12,13). Nonetheless, follow-up studies on adults indicate a shift to subclinical  hypothyroidism and hypothyroidism from euthyroidism (14,15,16). In the four-year follow-up study conducted by Radetti et al (8), the majority of euthyroid patients remained euthyroid, whereas those with subclinical hypothyroidism were observed to develop clinical hypothyroidism. Researchers suggest that TSH levels at the time of presentation are important in signaling the clinical course of the disease (15,16). Whickham, in a 20-year follow-up study, found the annual risk of spontaneous hypothyroidism as 0.35% (14). Moore et al (17) followed 17 children with euthyroid juvenile autoimmune thyroiditis for 52 months and found that only one required permanent treatment, and claimed that therapy does not affect the clinical prognosis in juvenile autoimmune thyroiditis. In another study conducted by Gruñeiro de Papendieck et al (18) in 16 children, nine of the children became euthyroid, two had subclinical hypothyroidism that remained unchanged, and five children shifted to hypothyroidism. In our study, 37 patients were receiving treatment at presentation and 64 were not under treatment. Overall, 36% of our 101 patients were euthyroid, 33% had subclinical hypothyroidism, and 17% had hypothyroidism. These rates, in respective order, were 24%, 33% and 43% in the study conducted by Gopalakrishnan et al (12) and 21%, 43% and 37% in the study conducted by de Vries et al (19). 

The majority of long-term follow-up studies including quantitative thyroid antibody measurements have been performed on a small sample of adults (20,21,22,23,24, 25,26,27). Mariotti et al (25) , in their study which comprised 77 hypothyroid and 21 euthyroid patients who had been on levothyroxine therapy for 12 to 18 months, found a significant decrease in the anti-TPO levels of 15 patients.  Romaldini et al (26) observed a 78% decrease in the anti-TPO levels and an 81% reduction in thyroid volume in 10 patients after six months of therapy. The researchers highlighted that treatment led to a significant decrease in the anti-Tg and anti-TPO values of patients with hypothyroidism, while only a non-significant decrease was noted in those with subclinical hypothyroidism. In their study conducted on 38 patients, Schmidt et al (27) reported a decrease in the anti-TPO values in the majority of patients receiving levothyroxine; however, these same authors reported negative anti-TPO titers in only 16% of the patients at the end of the 50-month follow-up period. They observed a fluctuation in the anti-TPO levels of 8% of the patients despite therapy, and consequently, the levels were increased in some patients. Hegedüs et al (28) found no significant decrease in anti-TPO values after two years of follow-up in a 13-patient study group. In this present study, the mean anti-Tg levels showed a significant decreased in the group receiving treatment. However, our sample size was too small to state that levothyroxine treatment decreased the anti-Tg levels.  We found a decrease in anti-Tg values in patients in both groups, regardless of therapy.  Padberg et al (22) conducted a study on 21 cases and found a decrease in the anti-TPO values of patients with euthyroid HT who were receiving treatment, while no decrease was observed in patients not receiving therapy. The decrease observed in anti-Tg values in the same study was not significant.

Despite the fact that growth retardation is one of the most common complaints at presentation in patients with HT, no abnormality in terms of height, weight and BMI SDS values was observed in the present study. This may be attributed to the fact that overall rate of hypothyroidism was 6.1% to 16.8% in this group of patients and that most of these patients were rendered euthyroid during the long-term follow-up.

In our study, due to the limited number of patients on levothyroxine treatment who had been followed by US, the findings pertaining to regression of the thyromegaly are not conclusive. Scarpa et al (29) studied 50 euthyroid non-goitrous children and adolescents with HT for 2 years by thyroid function tests and US and reported a lower thyroid volume SDS in the treatment group as compared to the controls. Svensson et al (30) reported that levothyroxine treatment significantly reduced the thyroid volume in pediatric patients with goitrous, hypothyroid and euthyroid HT. 

In conclusion, thyroid functions should be monitored periodically for hypothyroidism in all children and adolescents with HT, including those who are initially euthyroid or have subclinical hypothyroidism. Most patients who are not receiving treatment at the time of presentation may require treatment subsequently. Levothyroxine therapy may have beneficial effects on the clinical course of the disease and on antibody titers. Randomized controlled studies on large pediatric patient series are needed.
